# Metabolic alterations in children with environmental enteric dysfunction

**DOI:** 10.1038/srep28009

**Published:** 2016-06-13

**Authors:** Richard D. Semba, Michelle Shardell, Indi Trehan, Ruin Moaddel, Kenneth M. Maleta, M. Isabel Ordiz, Klaus Kraemer, Mohammed Khadeer, Luigi Ferrucci, Mark J. Manary

**Affiliations:** 1 Wilmer Eye Institute, Johns Hopkins University School of Medicine, Baltimore, MD 21287, USA; 2 National Institute on Aging, National Institutes of Health, Baltimore, MD 21224, USA; 3 Department of Pediatrics, Washington University in St. Louis, St. Louis, MO 63110 USA; 4 School of Public Health and Family Medicine, University of Malawi College of Medicine, Blantyre, Malawi; 5 Sight and Life,, CH-4002, Basel, Switzerland; 6 Johns Hopkins Bloomberg School of Public HealthBaltimore, MD 21205, USA

## Abstract

Environmental enteric dysfunction, an asymptomatic condition characterized by inflammation of the small bowel mucosa, villous atrophy, malabsorption, and increased intestinal permeability, is a major contributor to childhood stunting in low-income countries. Here we report the relationship of increased intestinal permeability with serum metabolites in 315 children without acute malnutrition, aged 12–59 months, in rural Malawi. Increased gut permeability was associated with significant differences in circulating metabolites that included lower serum phosphatidylcholines, sphingomyelins, tryptophan, ornithine, and citrulline, and elevated serum glutamate, taurine, and serotonin. Our findings suggest that environmental enteric dysfunction is characterized by alterations in important metabolites involved in growth and differentiation and gut function and integrity.

Environmental enteric dysfunction (EED) is highly prevalent in children younger than five years of age in rural sub-Saharan Africa, South America, and South Asia^1^,^2^. EED is strongly associated with childhood stunting^1^,^2^,^3^,^4^. Longitudinal studies of young children in The Gambia showed that >40% of linear growth failure is attributable to EED^4^. Stunting was associated with increased intestinal permeability but not with diarrhea history^5^. Close contact with animals, and more frequent geophagy are significant risk factors for EED in young children^1^,^2^,^6^. Chronic fecal-oral transmission of pathogenic bacteria, viruses, and parasites is considered a key factor underlying EED^1^,^2^. The pathological findings of EED include villous shortening, crypt hyperplasia, and lymphocytic infiltration^7^. With the breakdown of the intestinal barrier, there is translocation of bacteria and microbial products into the circulation, leading to chronic immune stimulation and the characteristic histopathology of EED^1^,^2^. Accordingly, immunohistochemical studies show T-cell infiltration and elevated proinflammatory cytokines such as tumor necrosis factor-α and interferon-γ in the lamina propria^7^.

Increased gut permeability could potentially affect the absorption and metabolism of amino acids, proteins, lipids, carbohydrates, and other nutrients^1^. However, this hypothesis has not been adequately investigated, and the underlying biological mechanisms by which EED could affect child health and linear growth are incompletely understood. This may explain why the many clinical trials that have been conducted with the aims of reducing gut permeability and improving linear growth among children in low-income countries have generally failed to both reduce gut permeability and improve child growth^2^. A better understanding of the basic biological mechanisms by which EED adversely impacts child health and growth may lead to more effective interventions against EED to reduce childhood stunting. We investigated the hypothesis that increased gut permeability is associated with concentrations of amino acids, glycerophospholipids, and sphingolipids by using a targeted metabolomics approach to characterize serum metabolites in children with and without EED in rural Malawi. We present data on young children from rural Malawi. Our results highlight specific metabolic alterations associated with increased gut permeability in young children with EED that are likely to affect growth and development.

## Results

### Study setting

We examined the relationship between gut permeability and serum metabolites in 315 children without acute malnutrition, aged 12–59 months, from six villages in rural Malawi. The villages where the children live are comprised largely of subsistence farm families who grow and consume maize as the staple crop. Water is acquired from boreholes or nearby streams. Homes are mostly of mud and thatch. Defecation generally occurs outdoors or in pit latrines. There is frequent contact between children and domestic animals such as chickens and goats. Electricity, running water, and other utilities are invariably absent in the homes. The environment of these rural villages in Malawi is a typical setting for EED. The characteristics of the children with and without EED in the study and demographic features of their household environment are shown in Table 1. There were nearly an equal number of boys and girls. The lactulose:mannitol (L:M) ratio, assessed in the dual-sugar absorption test, was used as the measure of gut permeability and intestinal absorption^8^. A higher L:M ratio indicates greater gut permeability. Children with an L:M ratio ≥ 0.15 were defined as having EED.^9^ Serum metabolites were measured using liquid chromatography-tandem mass spectrometry^10^. The 139 metabolites analyzed included 22 amino acids, 3 biogenic amines, 6 amino acid metabolites, 15 sphingolipids, 8 acylcarnitines, and 85 glycerophospholipids.

### Serum metabolites differ by gut permeability

**Figure 1:**
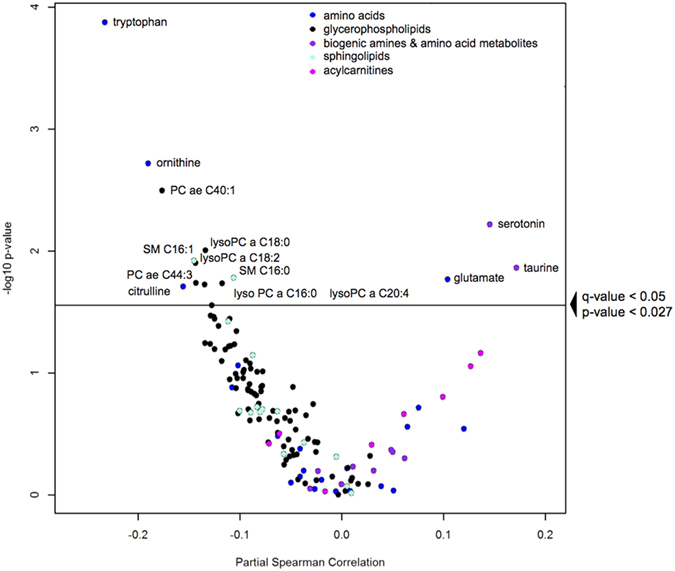
Volcano plot showing the relationship of partial Spearman correlations between the gut permeability (L:M ratio) and serum metabolites, adjusted for age, gender, and village. Horizontal line indicates significance at p-value of <0.027, which corresponds to a q-value < 0.05.

**Figure 2:**
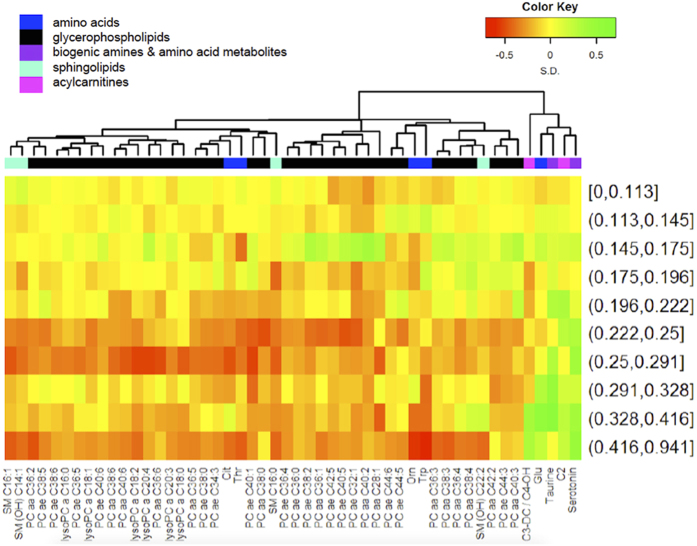
Heat map and hierarchical clustering of the 50 most significantly correlated metabolites with L:M ratio as continuous variable, after adjustment for age, gender, and village. L:M ratio is categorized in deciles. Z-scores shown for metabolites. Abbreviations for lipid nomenclature are described in the methods section. Amino acid abbreviations: Trp (tryptophan), Orn (ornithine), Glu (glutamate), Cit (citrulline).

**Figure 3:**
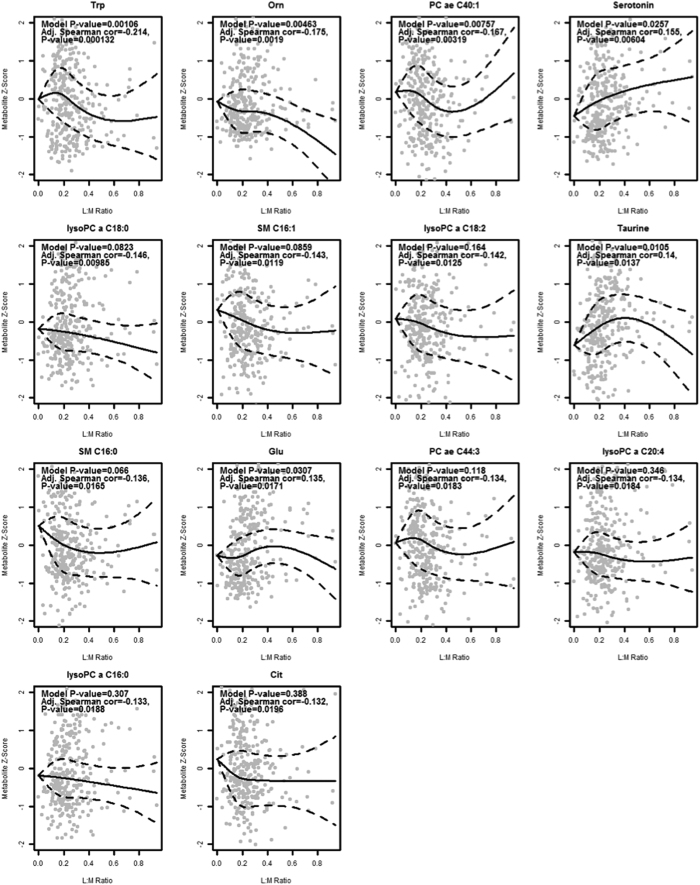
Scatterplots and fitted regression curves using non-linear cubic splines for serum metabolites with significant correlations with the L:M ratio. Broken lines indicate 95% confidence intervals. Data were modeled using linear mixed effects models that include age and sex and village as a random intercept. Correlation coefficients are shown. Abbreviations for lipid nomenclature are described in the methods section. Amino acid abbreviations: Trp (tryptophan), Orn (ornithine), Glu (glutamate), Cit (citrulline).

**Figure 4:**
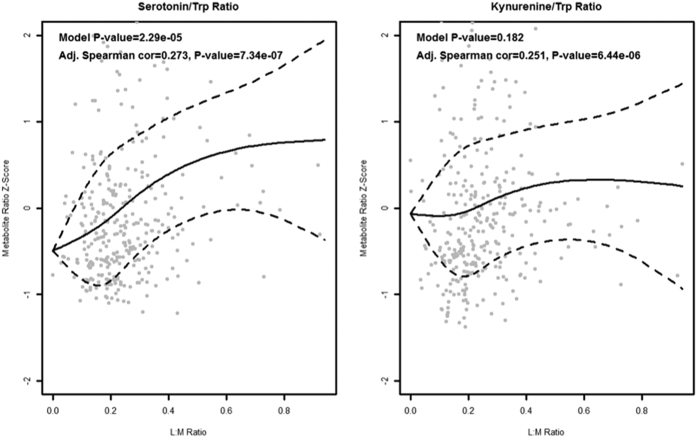
Scatterplots and fitted regression curves using non-linear cubic splines for serotonin/tryptophan ratio and kynurenine/tryptophan ratio with L:M ratio. Broken lines indicate 95% confidence intervals. Data were modeled using linear mixed effects models that include age and sex and village as a random intercept. Correlation coefficients are shown.

The relationship of gut permeability with all serum metabolites is summarized in a volcano plot in Fig. 1. There were 14 serum metabolites that were significantly related with gut permeability. Serum tryptophan, ornithine, citrulline, four lysophosphatidylcholines (lysoPC a C16:0, lysoPC a C18:0, lysoPC a C18:2, lysoPC a C20:4), two acyl-alkyl-phosphatidylcholines (PC ae C40:1, PC ae C44:3), and two sphingomyelins (SM C16:0, SM C16:1) were negatively correlated with gut permeability (L:M ratio). The general negative correlation of the different serum phosphatidylcholines and sphingomyelins with gut permeability as displayed on the left side of the volcano plot is quite evident. Serum glutamate, serotonin, and taurine were positively correlated with gut permeability. Tryptophan that is converted to serotonin or to kynurenine is no longer available for protein synthesis, thus, ratios of these two metabolites with tryptophan were calculated. The adjusted Spearman correlations between gut permeability and the serotonin/tryptophan and kynurenine/tryptophan ratio were 0.27 (*P* = 7.34 × 10^−7^) and 0.25 (*P* = 6.44 × 10^−6^), respectively. A summary heatmap of the top fifty serum metabolites by gut permeability and their clusters is shown in Fig. 2. We further investigated potential nonlinear relationships of the metabolites that were significantly correlated with gut permeability. Scatterplots and fitted regression curves (with 95% confidence intervals) using linear mixed-effects models to address within-village clustering with natural cubic splines (3 knots) are shown for the significant metabolites in Fig. 3 and for the serotonin/tryptophan and kynurenine/tryptophan ratios in Fig. 4. While nonlinearity is evident for some metabolites, inflection tends to occur at values of the L:M ratio where there are few data points and therefore uncertainty in estimation.

The age-, sex-, and village-adjusted Spearman correlations between gut permeability and serum metabolites are presented for amino acids, amino acid metabolites, and biogenic amines (Supplementary Table 1), sphingolipids and acylcarnitines (Supplementary Table 2), and glycerophospholipids (Supplementary Table 3). The correlations between gut permeability and serum metabolites ranged between −0.214 (tryptophan) to 0.155 (serotonin). More detailed heatmaps of serum amino acids, biogenic amines, amino acid metabolites, sphingolipids, acylcarnitines, and glycerophospholipids by gut permeability are shown in Supplementary Figs 1 and 2. Exploratory analyses showed that additional adjustment for height-for-age Z-score had little effect upon the relationship of the estimated correlations of serum metabolites with gut permeability. The relationships between urinary mannitol and metabolites were also explored. Only serum taurine and glutamate were associated with urinary mannitol (r = −0.173, *P *= 0.002; r = −0.157, *P *= 0.006, respectively). Unadjusted median (interquartile range) of serum metabolite concentrations in children with and without EED are shown for amino acids, biogenic amines, amino acid metabolites, sphingolipids, acylcarnitines, and glycerophospholipids in Supplementary Tables 4–6.

Multiple machine-learning classification algorithms were used to assess the ability of the 14 metabolites significantly correlated with L:M ratio to classify EED status (EED vs. no EED). We used the Super Learner^11^ algorithm to fit, internally cross-validate, and combine the results into a single classifier. Nine different classifiers were trained on a subset of the data and fit using data omitted for training via leave 10% out internal cross-validation. Next, a weighted-average of the estimates was computed to minimize mean-square error, and the weights themselves were estimated via leave 10% out internal cross-validation (See Supplementary Appendix for additional details). Five algorithms had positive weights (ridge regression^12^, generalized boosted models^13^, random forests^14^, multivariate adaptive regression splines implemented using the earth() function in R software version 3.2.0^15^,^16^, and Bayes generalized linear models). Supplementary Figure 3 and Supplementary Appendix Table 1 show that the metabolites have a modest ability to discriminate between children with and without EED (cross-validated Super Learner cross-validated area under the ROC curve 0.679, 95% confidence interval 0.610, 0.747). However, the final cross-validated Super Learner model was well calibrated showing little evidence of lack of fit (*P* = 0.70).

## Discussion

The present study shows that children with higher gut permeability, as measured by the L:M ratio, have alterations in serum metabolites that include lower serum phosphatidylcholines, lysophosphatidylcholines, and sphinogomyelins, lower serum tryptophan, ornithine, and citrulline, and higher serum glutamate, taurine, and serotonin. This is the first study to apply a targeted metabolomics approach to gain insight into the pathophysiology of EED. The results suggest that there may be abnormalities in several diverse metabolic pathways in children with increased gut permeability, including the CDP-choline or “Kennedy pathway” for synthesis of phosphatidylcholines, the synthesis of lysophosphatidylcholines and sphingomyelins, the tryptophan-serotonin pathway, the kynurenine pathway, and citrulline synthesis. Perturbations in these pathways and imbalance of metabolites could have potential adverse consequences for child health and growth. The individual pathways are discussed below.

Phosphatidylcholines, the dominant phospholipids in cell membranes and in the circulation, play a major role in cell proliferation, differentiation, and growth^17^. Phosphatidylcholines are essential for assembly and secretion of very low-density lipoproteins and high-density lipoproteins by the liver^18^. Phosphatidylcholines are the main active component of pulmonary surfactant and are vital to pulmonary function^19^. Phosphatidylcholines and lysophosphatidylcholines comprise >90% of the lipids in the protective mucus layer of the gastrointestinal tract^20^. Phosphatidylcholines are synthesized via the CDP-choline pathway in three enzymatic steps from dietary choline^17^. Phosphatidylcholines can also be synthesized via the phosphatidylethanolamine N-methyltransferase (PEMT) pathway. However, since the PEMT pathway accounts for ~30% of hepatic synthesis of phosphatidylcholines, it is only quantitatively important in the liver and cannot meet the body’s needs. Thus, choline is considered an essential nutrient^21^. The availability of phosphatidylcholine affects lysophosphatidylcholine, which is produced from the hydrolysis of phosphatidylcholine. Lysophosphatidylcholines are important constituents of biological membranes and bioactive phospholipids involved in inflammation^22^.

Since sphingomyelins are the dominant sphingolipids in membranes of mammalian cells, especially the outer leaflet, the endocytic recycling compartment, and the trans Golgi network, they may affect a large number of biological mechanisms. For example, sphingomyelins serve as a precursor to bioactive sphingolipids, are building blocks of lipid rafts, regulate plasma membrane homeostasis, and are involved in T cell activation and differentiation^23^,^24^,^25^. Sphingomyelins play a key role in the myelination of the central nervous system during the development of infants and young children^26^.

Tryptophan is an essential amino acid for protein synthesis as well as a precursor to the neurotransmitter serotonin. The major route for tryptophan metabolism is the kynurenine pathway in which tryptophan is oxidized by cleavage of the indole ring^27^. Tryptophan that is metabolized to serotonin or the kynurenine pathway is lost to protein synthesis^27^. Plasma tryptophan is decreased during inflammation or infection due to increased tryptophan oxidation^27^. The present study shows that the serotonin/tryptophan and kynurenine/tryptophan ratios are significantly correlated with gut permeability. Limitation of tryptophan availability for protein synthesis could potentially have adverse consequences for child growth. Dietary tryptophan limitation in Sprague Dawley rats resulted in severe growth restriction of young animals^28^. About 5% of dietary tryptophan is degraded to indole and other derivatives by bacteria in the gut lumen^29^. Possible mechanisms by which EED could affect tryptophan metabolism is through increased tryptophan degradation by altered gut microbiota, or through alterations in tryptophan transport by amino acid transporters^29^ in damaged enterocytes.

In contrast to the other amino acids that circulate in free form, about 80–90% of tryptophan is bound to albumin in the circulation, while the remaining free form can be transported across the blood-brain barrier^27^. Serotonin does not cross the blood-brain barrier, thus, tryptophan can give rise to two separate pools of serotonin in the brain and in the periphery, where it is mostly found in the gastrointestinal mucosa^29^. A limitation of the present study is that serum albumin was not measured. About 95% of total serotonin content in the human body is present in the gut, primarily in enterochromaffin cells of the intestinal mucosa^30^. Enterochromaffin cells act as local sensory transducers that respond to various chemical and mechanical stimuli in the gut^31^. Serotonin is synthesized from tryptophan through hydroxylation by tryptophan hydroxylase (TPH) followed by decarboxylation by aromatic acid decarboxylase (AADC). Serotonin mediates gastrointestinal functions that include peristalsis, secretion, vasodilation, and perception of pain or nausea^31^. Serotonin and one if its receptors, 5-HT3, are implicated as modulators of gastrointestinal inflammation in inflammatory bowel disease^31^, and the association with increased gut permeability suggests it may play a role in EED. Serotonin is removed from the intestinal mucosa by the serotonin-selective reuptake transporter (SERT). The underlying cause of elevated serum serotonin in children with EED is unclear. Possible biological mechanisms include upregulation of enzymes involved in serotonin synthesis, TPH and AADC, or a decrease in SERT expression during EED.

Serum glutamate concentrations were elevated in children with increased gut permeability. Glutamate, a non-essential amino acid, plays many important roles, such as providing a link between the tricarboxylic acid cycle and urea cycle in energy generation, serving in hepatic amino acid catabolism, being a precursor to glutathione, and acting as the primary fast excitatory neurotransmitter^32^,^33^. Glutamate, a main constituent of dietary protein, is a major oxidative fuel for the small intestine and is almost completely metabolized by the gut on first pass, presumably in the entrocytes^32^. Glutamate is also used for protein synthesis in the intestinal mucosa and can be used by enterocytes to produce aspartate, alanine, proline, ornithine, and citrulline^34^. Whether elevated serum glutamate arises from decreased utilization of glutamate by the gut, increased catabolism of amino acids such as arginine, ornithine, proline, histidine and glutamine, or other mechanisms in children with increased gut permeability is not known.

Taurine is an abundant free amino acid that plays diverse biological roles in membrane stabilization, cell volume regulation, mitochondrial function, growth and development, lung function, the conjugation of bile acids, and as an antioxidant^35^,^36^. Taurine is synthesized via the cysteine sulfinic acid pathway. The biological mechanisms that contribute to elevated serum taurine concentrations in children with EED are unclear.

Serum citrulline is considered to reflect total small bowel enterocyte mass since circulating citrulline is mainly produced by enterocytes^37^,^38^. Low circulating citrulline occurs in patients with short bowel syndrome, villous atrophy states, and Crohn’s disease^38^. Thus, serum citrulline has been proposed as a potential marker for EED^2^. Citrulline is synthesized in enterocytes via the conversion of glutamine or arginine to ornithine^39^. Citrulline is then produced from the conversion of ornithine by ornithine transcarbamylase^38^. The association of both low serum citrulline and ornithine with increased gut permeability may reflect reduced small bowel enterocyte mass.

The present study suggests several serum metabolites as candidate biomarkers for EED: citrulline, ornithine, glutamate, taurine, serotonin, tryptophan, serotonin/tryptophan ratio, kynurenine/tryptophan ratio, and phosphatidylcholines and sphingomyelins. However, the correlations between specific serum metabolites and gut permeability were modest, which may limit their use as biomarkers. Other blood biomarkers have been proposed as marker for gut permeability, including zonulin, anti-endotoxin core antibody, and soluble CD14^2^. Further studies are needed to determine the utility of selected serum metabolites as biomarkers of EED, including consideration of the specificity of the markers.

The present study is limited in that the findings cannot necessarily be generalized to all settings where children are at risk for EED. There may be differences in diet and environment that could potentially modify the relationship between increased gut permeability and circulating metabolites. Urinary lactulose and mannitol in the present study were measured using high performance liquid chromatography (HPLC). A recent analysis suggests that LC-MS/MS platforms using multiple reaction monitoring for measurement of urinary lactulose and mannitol is more accurate, although measurements using either HPLC or LC-MS/MS are highly correlated^40^. Another limitation is that other markers for EED were not measured in this study.

In conclusion, increased gut permeability is associated with alterations in important metabolites involving growth and differentiation and gut function and integrity. Further studies are needed to elucidate the relationship between metabolic alterations in EED and specific biological mechanisms, including insufficient dietary choline and tryptophan, the effects of increased gut permeability on metabolic pathways involving glutamine, serotonin, and taurine, and to corroborate the relationship of gut permeability with serum citrulline, ornithine, and serotonin/tryptophan and kynurenine/tryptophan ratios.

## Methods

### Study design and participants

We examined the relationship of EED with serum metabolites in a cross-sectional study of 315 children, aged 12–59 months, seen in six villages (Masika, Makhwira, Mitondo, Mibiza, Chamba, and Mayaka) in rural southern Malawi in 2011. Children were eligible for the study if they had no evidence of kwashiorkor, congenital or chronic disease, caretaker-reported diarrhea, or were under treatment for malnutrition. Children had anthropometry conducted by experienced field workers. Weight was measured to the nearest 5 g using a digital scale (Seca 344, Chino, CA). Height was measured to the nearest 0.1 cm using a rigid height board (Seca 417). The dual sugar permeability test was used as the standard non-invasive measure of gut integrity, as described in detail elsewhere^3^. The urinary L:M ratio was used as the measure of intestinal mucosal permeability^8^. Lactulose and mannitol were measured using high-performance liquid chromatography^3^. Chichewa-speaking Malawian research nurses obtained written and oral informed consent from each child’s caretaker before enrollment in the study. Community consent for the study also was obtained from the village chief and local health officials. The protocol for this study was approved by the College of Medicine Research Ethics Committee of the University of Malawi, the Human Research Protection Office of Washington University in St. Louis, and the Johns Hopkins School of Medicine Institutional Review Board. The methods were carried out in accordance with the approved guidelines.

### Measurement of serum metabolites

Venous blood was drawn by study nurses and doctors. Serum samples were processed, aliquoted, and snap frozen in liquid nitrogen in cryovials within 4 h of blood drawing. Cryovials were transferred to storage at −80 °C. Serum metabolites were measured using liquid chromatography tandem mass spectrometry (LC-MS/MS). Metabolites were extracted and concentrations are measured using the AbsoluteIDQ p180 kit (Biocrates Life Sciences AG, Innsbruck, Austria) following the manufacturers protocol for a 5500 QTrap (Sciex, Framingham, MA) mass spectrometer equipped with an electrospray ionization source, a Shimadzu CBM-20A command module, LC-20AB pump, and a Shimadzu SIL-20AC-HT autosampler, a CTO-10Ac column oven heater, and running with Analyst 1.5.2 software, as described in detail elsewhere^10^. Briefly, 10 μL of serum was pipetted onto a 96 well Biocrates kit. The samples were dried at room temperature (RT) for 30 min. 50 μL of 5% PITC reagent was added and incubated for 20 min and the plate was dried under nitrogen for 1 h. 300 μL of 5 mM ammonium acetate in methanol was added and incubated at RT on a shaker (450 rpm) for 30 min. The plate was centrifuged at 500 × g for 2 min and labeled; 50 μL of each sample was transferred to a 96 deep well LC plate, and 10 uL of each sample was transferred to the 96 deep well FIA plate. To the LC plate, 450 μl of 40% methanol (in HPLC grade water) was added. To the FIA plate, 490 μL of FIA running solvent was added. 10 μL was injected onto the Eclipse XDB C18, 3.5 μm, 3.0 × 100 mm with a Phenomenex C18 Security Guard Cartridge, 3.0 mm ID. The mobile phase consisted of solvent A (water containing 0.2% formic acid) and solvent B (acetonitrile containing 0.2% formic acid), with the following gradient: 0–0.5 min 0% B, 5.5 min: 95% B; 6.5 min: 95% B; 7.0 min: 0% B; 9.5 min: 0% B. LC plate evaluation of the samples was carried out using the MetIDQ software. The FIA plate was run with 20 μL injection directly onto the MS at a flow of 30 μL/min with water/acetonitrile (1:1) containing 0.2% formic acid as the mobile phase, with the following flow rate program: 0–1.6 min: 30 μL/min; 2.4 min: 200 μL/min; 2.80 min: 200 μL/min and 3.00 min: 30 μL/min. Concentrations were calculated using the Analyst/MetIDQ software and reported in μmol/L. The method measured 140 metabolites, including 22 amino acids, 3 biogenic amines, 6 amino acid metabolites, 15 sphingolipids, 8 acylcarnitines, and 85 glycerophospholipids (lyso-, diacyl-, and acyl-alkyl phosphatidylcholines). Glycerophospholipids are differentiated on the basis of ester and ether bonds in the glycerol moiety. Diacyl or “aa” indicates that fatty acids are bound with ester bonds at the sn-1 and sn-2 positions on the glycerol backbone. Acyl-alkyl or “ae” indicates that the fatty acid at the sn-1 position is bound with an ether bond. The total number of carbon atoms and double bonds in fatty acid chains is represented by “C x:y”, where x denotes the number of carbons and y denotes the number of double bonds. Phosphatidylcholine (PC), lysophosphatidylcholine (lysoPC), and sphingomyelin (SM), and hydroxysphingomyelin (SM [OH]) are used as abbreviations. Standard amino acid abbreviations are used in all figures. Acylcarnitines were designated with abbreviations: carnitine (C0), acetylcarnitine (C2), proprionylcarnitine (C3), butyrylcarnitine (C4), hydroxybutyrylcarnitine (C4-OH [C3-DC]), hexadecanoylcarnitine (C16), octadecenoylcarnitine (C18), and octadecadienylcarnitine (C18:1). Dimethylarginines were designated with abbreviations: total dimethylarginine (TDMA), symmetric dimethylarginine (SDMA), and asymmetric dimethylarginine (ADMA). The MS spectra were evaluated using Analyst/MetIDQ (Biocrates) software. Human serum samples spiked with standard metabolites were used to monitor the reproducibility of the assay. Serum metabolites that were below the limit of quantification were excluded from the data analyses. The inter-assay and intra-assay coefficients of variation ranged from 5% to 15% for nearly all analytes.

### Statistical analysis

Exploratory data analyses using histograms and boxplots were used to examine the distribution of the L:M ratio and serum metabolites. The L:M ratio is a measure of gut permeability. Spearman correlations of each metabolite with L:M ratio were estimated with and without adjustment for age, gender, and village. A false discovery rate approach was used to correct for multiple testing; the bootstrap method was used to compute q-values^41^,^42^,^43^. This analysis was carried out using the pcor.test() function in the ppcor package in R software version 3.2.0. Classification modeling using nine machine-learning algorithms was used to examine the ability of the 14 metabolites that were significantly correlated with L:M ratio to classify EED status (L:M ≥ 0.15 versus L:M < 0.15). SuperLearner^11^, an ensembling approach to machine learning, implemented multiple individual machine-learning algorithms (e.g., logistic regression). The cross-validated results from the individual machine-learning algorithms were combined via weighted average to minimize a cross-validated mean squared error (and potentially fit the data better than each individual algorithm). Details of SuperLearner, including computation of estimates, cross-validation, and metrics to assess discrimination and calibration, are described in detail in the Supplementary Appendix. Classification modeling was carried out using the SuperLearner() and CV.SuperLearner() functions in the SuperLearner package in R software version 3.2.0^44^.

## Additional Information

**How to cite this article**: Semba, R. D. *et al*. Metabolic alterations in children with environmental enteric dysfunction. *Sci. Rep.* **6**, 28009; doi: 10.1038/srep28009 (2016).

## Supplementary Material

Supplementary Information

## Figures and Tables

**Table 1:** Characteristics of the study population.

Characteristic^1^		No EED^2^ (n = 68)	EED^2^ (n = 247)	*P*^3^
Age, months		37.9 (10.5)	33.7 (12.0)	0.006
Female, %		48.5	49.8	0.96
Weight-for-height Z-score		0.3 (1.0)	0.2 (0.9)	0.53
Height-for-age Z-score		−2.4 (1.3)	−2.3 (1.3)	0.69
Stunted, %		63.2	61.1	0.75
Caretaker is mother, %		97.0	95.1	0.50
Father is alive, %		89.7	97.2	0.008
Siblings, n		3.8 (1.7)	3.7 (1.7)	0.73
Individuals that sleep in same room as child, n		3.3 (1.7)	3.4 (1.4)	0.61
Home with a metal roof, %		27.9	17.4	0.05
Family owns bicycle, %		58.8	61.9	0.64
Animals sleep in house, %		44.1	35.2	0.18
Water from a clean source, %		82.3	66.0	0.009
Child uses pit latrine, %		76.5	66.0	0.18
Village, %	Chamba	10.4	14.2	<0.001
	Makwhira	4.5	6.1	
	Masika	14.9	42.9	
	Mayaka	38.8	22.7	
	Mbiza	22.4	11.7	
	Mitondo	9.0	2.4	

^1^Means (SD) or %.

^2^No EED (L:M < 0.15), EED (L:M ≥ 0.15).

^3^Students t-test for continuous variables or chi-square test for categorical variables.
