# Supplementary material for: Metabolic alterations in children with environmental enteric dysfunction
Source: Sci Rep. 2016 Jun 13;6:28009. doi: 10.1038/srep28009 (PMC4904796; doi:10.1038/srep28009)

Metabolic alterations in children with environmental enteric dysfunction

Richard D. Semba, Michelle Shardell, Indi Trehan, Ruin Moaddel,

Kenneth M. Maleta, M. Isabel Ordiz, Klaus Kraemer, Mohammed Khadeer,

Luigi Ferrucci & Mark J. Manary

Supplementary information

Supplementary Tables 1-6

Supplementary Appendix

Supplementary Figures 1-3

Supplementary Table 1. Spearman correlations of serum amino acids, biogenic amines, and amino acid metabolites with gut permeability (L:M ratio), adjusted by age, gender, and village

**Analyte**	***r***	***P***	**Analyte**	***r***	***P***
Tryptophan	-0.214	0.0001*	Alanine	0.006	0.92
Isoleucine	-0.030	0.60	Aspartic acid	-0.018	0.75
Leucine	-0.005	0.93	Glutamate	0.135	0.017*
Valine	-0.011	0.85	Citrulline	-0.132	0.019*
Methionine	-0.005	0.94	Ornithine	-0.175	0.002*
Histidine	0.074	0.19	Alpha-aminoadipic acid	-0.008	0.88
Phenylalanine	-0.027	0.63	Kynurenine	0.031	0.58
Threonine	-0.098	0.09	Creatinine	0.039	0.50
Lysine	-0.015	0.79	Spermine	-0.027	0.64
Asparagine	-0.018	0.75	Putrescine	0.028	0.63
Glutamine	-0.046	0.42	Serotonin	0.155	0.006*
Arginine	-0.056	0.33	Taurine	0.143	0.013*
Glycine	0.021	0.71	Total dimethylarginine	0.014	0.82
Proline	0.062	0.27	Symmetric dimethylarginine	0.045	0.43
Serine	0.061	0.28	Asymmetric dimethylarginine	0.044	0.44

Tyrosine	-0.086	0.13	

*Significant at q-value < 0.05 and p-value < 0.027

Supplementary Table 2. Spearman correlations of serum sphingolipids[1] and acylcarnitines with gut permeability (L:M ratio), adjusted by age, gender, and village

**Analyte**	***r***	***P***	**Analyte**	***r***	***P***
SM (OH) C14:1	-0.118	0.037	SM C24:1	-0.042	0.46
SM (OH) C16:1	-0.051	0.37	SM C26:0	0.003	0.96
SM (OH) C22:1	-0.072	0.21	SM C26:1	0.011	0.85
SM (OH) C22:2	-0.103	0.07	Carnitine (C0)	0.070	0.22
SM (OH) C24:1	-0.072	0.21	Acetylcarnitine (C2)	0.097	0.08
SM C16:0	-0.136	0.016*	Propionylcarnitine (C3)	0.081	0.16
SM C16:1	-0.143	0.011*	Butyrylcarnitine (C4)	0.049	0.38
SM C18:0	-0.075	0.18	Hydroxybutyrylcarnitine (C4-OH [C3-DC])	0.105	0.07
SM C18:1	-0.072	0.20	Hexadecanoylcarnitine (C16)	-0.055	0.38
SM C20:2	-0.073	0.19	Octadecenoylcarnitine (C18)	-0.005	0.93
SM C22:3	-0.072	0.20	Octadecadienylcarnitine (C18:1)	-0.059	0.31
SM C24:0	-0.040	0.48			

[1]Abbreviations for lipid nomenclature are described in the Methods section.

*Significant at q-value < 0.05 and p-value < 0.027

Supplementary Table 3. Spearman correlations of serum glycerophospholipids[1] with gut permeability (L:M ratio), adjusted by age, gender, and village

**Analyte**	***r***	***P***	**Analyte**	***r***	***P***
LysoPC a C16:0	-0.133	0.018*	PC aa C42:0	-0.077	0.18
LysoPC a C16:1	-0.073	0.20	PC aa C42:1	-0.077	0.18
LysoPC a C17:0	-0.044	0.44	PC aa C42:2	-0.093	0.10
LysoPC a C18:0	-0.146	0.009*	PC aa C42:4	-0.068	0.23
LysoPC a C18:1	-0.125	0.027	PC aa C42:5	-0.040	0.48
LysoPC a C18:2	-0.142	0.012*	PC aa C42:6	-0.071	0.22
LysoPC a C20:3	-0.119	0.035	PC ae C30:1	0.005	0.92
LysoPC a C20:4	-0.134	0.018*	PC ae C30:2	-0.084	0.13
LysoPC a C24:0	-0.051	0.37	PC ae C32:1	-0.086	0.13
LysoPC a C26:0	0.013	0.82	PC ae C32:2	-0.082	0.15
LysoPC a C26:1	0.001	0.99	PC ae C34:0	0.013	0.81
LysoPC a C28:0	0.041	0.48	PC ae C34:1	-0.018	0.75
LysoPC a C28:1	-0.020	0.72	PC ae C34:2	-0.037	0.51
PC aa C24:0	-0.023	0.68	PC ae C34:3	-0.095	0.10
PC aa C28:1	-0.020	0.72	PC ae C36:0	-0.091	0.11

PC aa C30:0	-0.066	0.24	PC ae C36:1	-0.042	0.46
PC aa C30:2	-0.074	0.19	PC ae C36:2	-0.014	0.80
PC aa C32:0	-0.041	0.47	PC ae C36:3	-0.054	0.35
PC aa C32:1	-0.033	0.56	PC ae C36:4	-0.121	0.03
PC aa C32:2	-0.019	0.74	PC ae C36:5	-0.095	0.09
PC aa C32:3	-0.045	0.43	PC ae C38:0	-0.099	0.08
PC aa C34:1	-0.058	0.31	PC ae C38:1	-0.084	0.14
PC aa C34:2	-0.082	0.15	PC ae C38:2	-0.014	0.80
PC aa C34:3	-0.048	0.39	PC ae C38:3	-0.060	0.29
PC aa C34:4	-0.085	0.14	PC ae C38:4	-0.071	0.21
PC aa C36:0	-0.072	0.21	PC ae C38:5	-0.100	0.08
PC aa C36:1	-0.116	0.04	PC ae C38:6	-0.091	0.11
PC aa C36:2	-0.119	0.04	PC ae C40:1	-0.167	0.003*
PC aa C36:3	-0.094	0.10	PC ae C40:2	-0.051	0.37
PC aa C36:4	-0.090	0.11	PC ae C40:3	-0.066	0.25
PC aa C36:5	-0.114	0.04	PC ae C40:4	-0.073	0.20
PC aa C36:6	-0.100	0.08	PC ae C40:5	-0.108	0.06
PC aa C38:0	-0.088	0.12	PC ae C40:6	-0.107	0.06
PC aa C38:1	-0.030	0.60	PC ae C42:1	-0.086	0.13

PC aa C38:3	-0.105	0.06	PC ae C42:2	-0.068	0.23
PC aa C38:4	-0.096	0.09	PC ae C42:3	-0.084	0.14
PC aa C38:5	-0.066	0.25	PC ae C42:4	-0.021	0.71
PC aa C38:6	-0.094	0.09	PC ae C42:5	-0.087	0.13
PC aa C40:2	-0.094	0.09	PC ae C44:3	-0.134	0.018*
PC aa C40:3	-0.108	0.06	PC ae C44:4	-0.052	0.37
PC aa C40:4	-0.067	0.24	PC ae C44:5	-0.087	0.13
PC aa C40:5	-0.053	0.35	PC ae C44:6	-0.106	0.06
PC aa C40:6	-0.120	0.03			

[1]Abbreviations for lipid nomenclature are described in the Methods section.

*Significant at q-value < 0.05 and p-value < 0.027

Supplementary Table 4. Serum amino acids, biogenic amines, and amino acid metabolite concentrations in children with and without EED

Analyte in µmol/L	No EED (n = 68)		EED (n = 247)		*P*[1]	Analyte in µmol/L	No EED (n = 68)		EED (n = 247)		*P*[1]
	Median	IQR[2]	Median	IQR			Median	IQR	Median	IQR	
Tryptophan	36.5	20.6, 51.8	36.7	25.2, 50.8	0.66	Alanine	465.7	395.5, 592.1	492.6	418.6, 580.2	0.42
Isoleucine	51.1	44.6, 60.2	58.6	49.2, 70.0	0.004	Aspartic acid	54.4	47.3, 68.3	56.7	42.3, 70.3	0.82
Leucine	127.4	100.5, 150.2	138.2	116.4, 167.3	0.003	Glutamate	176.9	140.6, 218.8	251.7	156.9, 334.7	<0.0001
Valine	130.9	114.6, 157.3	146.4	125.0, 176.6	0.002	Citrulline	20.4	16.7, 26.3	20.3	16.1, 26.6	0.45
Methionine	20.6	17.9, 24.8	23.8	19.4, 28.7	0.003	Ornithine	69.1	56.1, 90.2	74.9	54.3, 116.5	0.16
Histidine	83.4	70.1, 97.4	92.6	75.9, 112.4	0.001	Alpha-aminoadipic acid	1.14	0.81, 9.66	1.07	0.70, 3.17	0.58
Phenylalanine	81.9	70.6, 94.3	85.5	75.5, 99.5	0.05	Kynurenine	2.61	2.02, 3.16	2.82	2.09, 3.58	0.08
Threonine	89.7	72.1, 105.4	89.1	69.6, 118.1	0.76	Creatinine	21.8	17.7, 25.4	21.3	17.7, 25.1	0.72
Lysine	127.8	108.8, 147.7	147.5	112.8, 185.0	0.007	Spermine	0.09	0.06, 0.14	0.08	0.05, 0.13	0.05
Asparagine	54.4	47.3, 68.3	56.7	42.3, 70.3	0.83	Putrescine	0.12	0.07, 0.17	0.13	0.07, 0.19	0.59
Glutamine	523.4	442.5, 642.5	535.3	451.2, 627.0	0.71	Serotonin	1.14	0.72, 1.72	1.40	0.96, 1.98	0.02
Arginine	87.4	71.4, 103.9	82.6	67.7, 97.9	0.26	Taurine	152.1	113.7, 179.8	182.7	150.9, 226.9	<0.0001

Glycine	304.4	255.1, 362.8	327.8	265.0, 397.5	0.07	Total dimethylarginine	1.25	0.94, 1.46	1.20	0.90, 1.55	0.97
Proline	179.7	156.2, 221.1	191.5	163.3, 226.4	0.38	Symmetric dimethylarginine	0.49	0.16, 0.73	0.42	0.09, 0.70	0.28
Serine	143.3	129.3, 161.6	165.8	138.0, 208.1	0.0002	Asymmetric dimethylarginine	0.88	0.72, 1.03	0.92	0.75, 1.13	0.22
Tyrosine	60.8	48.3, 71.9	61.1	52.0, 72.9	0.60						

[1]Wilcoxon rank-sum test

[2]Interquartile range

Supplementary Table 5. Serum sphingomyelin[1] and acylcarnitine concentrations in children with and without EED

Analyte in µmol/L	No EED (n = 68)		EED (n = 247)		P^2	Analyte in µmol/L	No EED (n = 68)		EED (n = 247)		P^2
	Median	IQR[3]	Median	IQR			Median	IQR	Median	IQR	
SM (OH) C14:1	2.34	1.95, 2.78	2.23	1.94, 2.72	0.45	SM C24:1	38.5	32.9, 43.2	39.3	32.9, 44.5	0.69
SM (OH) C16:1	2.02	1.68, 2.25	2.04	1.68, 2.35	0.78	SM C26:0	0.20	0.16, 0.25	0.19	0.017, 0.23	0.61
SM (OH) C22:1	7.50	6.11, 9.04	7.38	6.25, 8.69	0.84	SM C26:1	0.28	0.23, 0.34	0.27	0.22, 0.31	0.19
SM (OH) C22:2	4.69	3.90, 5.47	4.69	3.96, 5.38	0.97	Carnitine (C0)	20.5	17.4, 26.6	22.3	18.5, 28.4	0.05
SM (OH) C24:1	0.87	0.71, 1.08	0.85	0.72, 1.02	0.36	Acetylcarnitine (C2)	3.36	2.80, 4.35	4.03	2.87, 6.79	0.003
SM C16:0	96.8	79.6, 115.0	88.0	77.0, 105.2	0.08	Proprionylcarnitine (C3)	0.46	0.35, 0.57	0.50	0.39, 0.65	0.07
SM C16:1	9.05	7.24, 11.1	8.60	7.42, 10.4	0.21	Butyrylcarnitine (C4)	0.23	0.17, 0.31	0.29	0.19, 0.36	0.01
SM C18:0	17.7	15.0, 22.8	18.1	14.1, 22.5	0.64	Hydroxybutyrylcarnitine (C4-OH [C3-DC])	0.37	0.27, 0.48	0.38	0.28, 0.48	0.80
SM C18:1	6.11	5.10, 7.92	6.27	5.35, 7.46	0.99	Hexadecanoylcarnitine (C16)	0.19	0.16, 0.24	0.21	0.16, 0.26	0.05
SM C20:2	0.59	0.49, 0.70	0.54	0.44, 0.66	0.05	Octadecenoylcarnitine (C18)	0.12	0.10, 0.16	0.14	0.10, 0.17	0.03
SM C22:3	5.79	4.73, 7.00	5.65	4.45, 6.79	0.29	Octadecadienylcarnitine	0.10	0.08, 0.12	0.11	0.08, 0.13	0.17

						(C18:1)					
SM C24:0	20.1	17.5, 24.8	20.8	17.9, 23.8	0.99						

[1]Abbreviations for lipid nomenclature are described in the Methods section.

[2]Wilcoxon rank-sum test

[3]Interquartile range

Supplementary Table 6. Serum glycerophospholipids[1] in children with and without EED

Analyte in µmol/L	No EED (n = 68)		EED (n = 247)		P^2	Analyte in µmol/L	No EED (n = 68)		EED (n = 247)		P^2
	Median	IQR[3]	Median	IQR			Median	IQR	Median	IQR	
LysoPC a C16:0	133.6	110.2, 174.6	157.0	108.5, 226.9	0.02	PC aa C42:0	0.73	0.60, 0.87	0.64	0.52, 0.77	0.002
LysoPC a C16:1	3.01	2.34, 3.94	3.21	2.34, 4.30	0.52	PC aa C42:1	0.42	0.34, 0.49	0.38	0.30, 0.46	0.02
LysoPC a C17:0	1.99	1.63, 2.80	2.61	1.81, 3.67	0.001	PC aa C42:2	0.26	0.21, 0.29	0.23	0.17, 0.28	0.02
LysoPC a C18:0	41.8	35.7, 50.1	49.4	34.8, 75.1	0.01	PC aa C42:4	0.23	0.20, 0.29	0.23	0.19, 0.29	0.59
LysoPC a C18:1	20.3	15.5, 24.3	21.2	16.4, 25.9	0.19	PC aa C42:5	0.41	0.34, 0.47	0.41	0.33, 0.50	0.88
LysoPC a C18:2	21.8	17.7, 30.5	21.6	16.8, 27.0	0.45	PC aa C42:6	0.53	0.43, 0.61	0.47	0.40, 0.61	0.12
LysoPC a C20:3	2.41	1.73, 3.04	2.51	1.83, 3.37	0.30	PC ae C30:1	0.19	0.13, 0.26	0.18	0.13, 0.24	0.54
LysoPC a C20:4	9.68	7.97, 12.40	10.81	8.28, 14.67	0.05	PC ae C30:2	0.07	0.06, 0.09	0.06	0.05, 0.08	0.019
LysoPC a C24:0	0.41	0.34, 0.48	0.41	0.34, 0.49	0.78	PC ae C32:1	2.40	1.96, 3.26	2.16	1.79, 2.73	0.009
Lyso PC a C26:0	0.56	0.42, 0.71	0.52	0.40, 0.66	0.29	PC ae C32:2	0.47	0.39, 0.59	0.43	0.36, 0.50	0.006
LysoPC a C26:1	0.30	0.24, 0.37	0.28	0.34, 0.36	0.40	PC ae C34:0	0.80	0.67, 1.14	0.82	0.66, 1.06	0.73
LysoPC a C28:0	0.42	0.34, 0.49	0.45	0.38, 0.54	0.08	PC ae C34:1	6.58	4.81, 8.59	5.62	4.43, 7.10	0.02
LysoPC a C28:1	0.47	0.41, 0.54	0.48	0.40, 0.56	0.65	PC ae C34:2	5.33	4.14, 6.64	4.98	4.28, 5.82	0.13
PC aa C24:0	0.19	0.15, 0.23	0.19	0.16. 0.24	0.52	PC ae C34:3	4.24	3.43, 4.98	3.91	3.17, 4.83	0.14

PC aa C28:1	1.07	0.84, 1.33	1.09	0.86, 1.66	0.41	PC ae C36:0	0.61	0.50, 0.71	0.54	0.45, 0.64	0.06
PC aa C30:0	2.54	1.73, 3.57	2.37	1.80, 3.33	0.65	PC ae C36:1	4.06	3.16, 5.05	3.57	2.73, 4.47	0.03
PC aa C30:2	0.43	0.33, 0.55	0.39	0.29, 0.52	0.17	PC ae C36:2	6.32	4.91, 8.25	5.82	4.81, 7.55	0.23
PC aa C32:0	14.5	10.4, 17.6	12.2	10.2, 15.3	0.04	PC ae C36:3	3.64	2.84, 4.33	3.34	2.89, 3.95	0.16
PC aa C32:1	10.6	6.0, 16.3	7.4	4.8, 12.1	0.003	PC ae C36:4	12.4	10.4, 15.4	11.5	9.79, 14.7	0.31
PC aa C32:2	1.80	1.24, 2.29	1.76	1.29, 2.33	0.83	PC ae C36:5	10.1	7.7, 11.4	9.6	7.9, 12.6	0.64
PC aa C32:3	0.24	0.20, 0.29	0.24	0.20, 0.28	0.45	PC ae C38:0	1.05	0.87, 1.33	1.01	0.84, 1.22	0.20
PC aa C34:1	145.4	104.5, 173.6	118.1	93.4, 147.6	0.003	PC ae C38:1	0.42	0.29, 0.53	0.35	0.25, 0.45	0.008
PC aa C34:2	214.4	182.2, 245.7	198.1	168.0, 227.0	0.03	PC ae C38:2	1.44	1.12, 1.97	1.26	1.00, 1.64	0.006
PC aa C34:3	6.85	5.31, 8.77	5.63	3.99, 7.67	0.001	PC ae C38:3	2.14	1.77, 2.60	2.04	1.64, 2.52	0.33
PC aa C34:4	0.81	0.65, 1.00	0.85	0.66, 1.12	0.29	PC ae C38:4	9.37	8.37, 11.09	9.33	8.02, 11.06	0.68
PC aa C36:0	1.91	1.54, 2.57	1.80	1.33, 2.34	0.10	PC ae C38:5	13.6	10.8, 16.2	13.1	11.2, 15.5	0.57
PC aa C36:1	32.0	25.5, 38.8	27.3	22.6, 34.3	0.001	PC ae C38:6	4.73	3.72, 5.85	4.55	3.86, 5.92	0.91
PC aa C36:2	135.6	115.4, 150.7	127.4	107.4, 145.0	0.03	PC ae C40:1	0.76	0.62, 0.94	0.70	0.52, 0.85	0.03
PC aa C36:3	68.8	55.8, 82.9	65.7	52.6, 74.8	0.14	PC ae C40:2	0.70	0.57, 0.85	0.66	0.54, 0.81	0.23
PC aa C36:4	137.8	115.4, 150.7	132.4	111.9, 151.5	0.37	PC ae C40:3	0.65	0.54, 0.75	0.61	0.51, 0.73	0.22
PC aa C36:5	6.95	5.12, 8.70	5.85	4.47, 8.04	0.01	PC ae C40:4	1.83	1.62, 2.12	1.76	1.53, 2.07	0.18
PC aa C36:6	0.47	0.39, 0.58	0.49	0.38, 0.65	0.47	PC ae C40:5	2.57	2.21, 2.89	2.41	2.11, 2.85	0.07
PC aa C38:0	2.43	2.15, 3.22	2.27	1.86, 2.92	0.08	PC ae C40:6	3.48	2.96, 4.01	3.40	2.86. 4.15	0.59

PC aa C38:1	0.79	0.54, 1.04	0.71	0.48, 0.97	0.29	PC ae C42:1	0.64	0.56, 0.74	0.57	0.45 0.71	0.007
PC aa C38:3	34.1	29.7, 41.8	34.0	28.4, 40.7	0.45	PC ae C42:2	0.42	0.35, 0.50	0.38	0.29, 0.48	0.09
PC aa C38:4	93.7	82.9, 108.8	98.6	79.0, 113.3	0.99	PC ae C42:3	0.48	0.40, 0.57	0.44	0.37, 0.55	0.11
PC aa C38:5	33.7	28.2, 38.5	34.0	28.4, 40.7	0.45	PC ae C42:4	0.63	0.52, 0.72	0.60	0.51, 0.70	0.48
PC aa C38:6	56.6	45.9, 68.4	56.1	43.8, 69.9	0.80	PC ae C42:5	1.54	1.37, 1.83	1.52	1.35, 1.73	0.15
PC aa C40:2	0.31	0.28, 0.37	0.28	0.22, 0.36	0.05	PC ae C44:3	0.27	0.23, 0.31	0.25	0.21, 0.30	0.07
PC aa C40:3	0.57	0.47, 0.64	0.52	0.41, 0.62	0.06	PC ae C44:4	0.32	0.27, 0.36	0.30	0.26, 0.34	0.06
PC aa C40:4	4.51	3.67, 5.63	4.19	3.43, 5.26	0.17	PC ae C44:5	1.15	1.00, 1.30	1.09	0.91, 1.27	0.16
PC aa C40:5	9.64	7.98, 11.66	9.57	7.88, 11.71	0.94	PC ae C44:6	1.66	1.50, 1.94	1.62	1.36, 1.88	0.06
PC aa C40:6	24.9	19.2, 29.9	24.5	19.4, 30.1	0.67						

[1]Abbreviations for lipid nomenclature are described in the Methods section.

[2]Wilcoxon rank-sum test

[3]Interquartile range

Supplementary Appendix

SuperLearner[1] is an ensembling approach to machine learning that involves two steps. First, multiple individual machine-learning classification algorithms (e.g., logistic regression) are implemented. Second, cross-validated results from the individual machine-learning algorithms are combined via weighted average to minimize a cross-validated mean squared error (and potentially fit the data better than each individual algorithm). In the first step, only a portion of the data is used to train each algorithm. The second step combines estimates using the data that were left out of the training data, producing a cross-validated estimate. Cross-validated SuperLearner additionally leaves out a portion of data in the second step to produce cross-validated estimates of the weights. The idea behind SuperLearner is that no one machine-learning algorithm is optimal for all datasets. Performance of an algorithm depends on the underlying true functional form linking the features (e.g., metabolites) to the outcome (e.g., EED), which is unknown in practice. Thus, SuperLearner allows the researcher to try multiple machine-learning algorithms and optimally combines the results to find the lowest mean squared error. A schematic for SuperLearner can be found in the paper by Rose[2].

In the current study, we implemented SuperLearner so that it splits the data into 10 mutually exclusive and collectively exhaustive groups. We ensured that the proportions with and without EED were nearly identical in each group. We ran multiple machine-learning algorithms 10 times, each using 9/10ths of the data (the training data) with a different group omitted each time (test data). Estimated models of each machine-learning algorithm were then applied to the omitted group ($1/10^{th}$ of the data) to compute the cross-validated (CV) estimated probability of EED; i.e., leave 10% out cross validation. It is important to stress that the estimated

probabilities were derived from data that were not used to train the algorithms. The final Super Learner estimate involved computing the weighted average of the CV estimates of the individual machine-learning algorithms that minimized the Brier score (equivalent to mean-square error). The weighting was carried out using non-negative least squares regression of EED on cross-validated probabilities of EED estimated from each individual machine-learning algorithm.

To further cross-validate the performance of the final Super Learner estimates (i.e., the coefficients, or weights, of the weighted average), we performed CV Super Learner, which splits the data into 10 mutually exclusive and collectively exhaustive groups, and performs Super Learner 10 times using 9/10ths of the data and leaves out a different 1/10th set each time. The final CV estimate was computed in the test data. We carried out Super Learner using the SuperLearner() and CV.SuperLearner() function in the SuperLearner package in R version 3.2.0[3]. We selected 9 individual machine-learning algorithms: 1) glmnet() in the glmnet package with alpha=1 (least absolute shrinkage and selection operator [LASSO] regularization)[4], 2) glmnet() with alpha=0 (ridge regression regularization)[4], 3) glmnet() with alpha=0.5 (elastic net regularization, which is a hybrid of LASSO and ridge regression regularization)[4], 4) gbm() in the gbm package (generalized boosted regression)[4,5] with 10,000 trees and interaction depth = 2, 5) randomForest() in the randomForest package (random forest ensemble of classification and regression trees) with 1,000 trees[6], 6) ipredbagg() in the ipred() package (bootstrap aggregation [BAGGing] of classification and regression trees)[6] with 100 replicates, 7) polymars() in the polspline package (adaptive regression using piecewise linear splines)[8,9], 8) earth() in the earth package[10] (another implementation of adaptive regression using piecewise linear splines)[11], and 9) bayesglm() in the arm package (main-effects Bayesian logistic regression) with a Cauchy prior with scale=2.5.

We computed multiple metrics to assess the cross-validated (i.e., in testing data not used to fit the models) performance of CV Super Learner, Super Learner, and the individual machine-learning algorithms (Supplementary Appendix Table 1). We computed the ROC curves (Supplementary Figure 3), and measures of discrimination including CV R2 and area under the ROC curve (AUC) with 95% confidence intervals. We also assessed model calibration using Brier scores, means (i.e., probabilities) of true EED status within strata of estimated probabilities, and the Hosmer-Lemeshow goodness-of-fit chi-square test[12] where p-value <0.05 is considered significant evidence of lack of fit.

References for Supplementary Appendix 1

1.van der Laan, M. J., Polley, E. C. & Hubbard, A. E. Super learner. *Stat. Appl. Genet. Mol. Biol.* **6**, Article 25 (2007).

2. Rose, S. Mortality risk score prediction in an elderly population using machine learning. *Am. J. Epidemiol.* **177**, 443-452 (2013).

3. Polley, E. C. & van der Laan, M. J. SuperLearner: Super Learner Prediction, Package Version 2.0-15. Vienna, Austria: R Foundation for Statistical Computing (2015).

4. Friedman, J., Hastie, T. & Tibshirani, R. Regularization paths for generalized linear models via coordinate descent. *J. Stat. Software* **33**, 1-22 (2010).

5. Friedman, J. H. Greedy function approximation: a gradient boosting machine. *Ann. Stat.* **29**, 1189-1232 (2001).

6. Breiman, L. Random forests. *Machine Learning* **45**, 5-32 (2001).

7. Breiman, L. Bagging predictors. *Machine Learning* **24**, 123-140 (1996).

8. Stone, C. J., Hansen, M., Kooperberg, C. & Truong, Y. K. The use of polynomial splines and their tensor products in extended linear modeling (with discussion). *Ann. Stat.* **25**, 1371–1470 (1997).

9. Kooperberg, C. *polspline: Polynomial Spline Routines, Package Version 1.1-2*. Vienna, Austria: R Foundation for Statistical Computing (2015).

10. Milborrow, S. *Earth: Multivariate Adaptive Regression Spline Models, Package Version 4.4-3.* Vienna, Austria: R Foundation for Statistical Computing (2015).

11. Friedman, J. H. Multivariate adaptive regression splines (with discussion) *Ann. Stat.* **19**, 1–141 (1991).

12. Hosmer, D. W., Hosmer, T., le Cessie, S. & Lemeshow, S. A comparison of goodness-of-fit tests for the logistic regression model. *Stat. Med.* **16**, 965-980 (1997).

Supplementary Appendix Table 1.

Metrics for discrimination and calibration of classification modeling using the Super Learner algorithm

Algorithm	Coefficients[a]	Cross Validated[b] Measures of Calibration and Discrimination				Stratified Means[b,c]						Hosmer-Lemeshow p-value
		Brier Score	R^2	AUC	AUC 95% CI	0 to <0.6	0.6 to <0.7	0.7 to <0.8	0.8 to <0.85	0.85 to <0.9	<0.9 to 1.0	
CV Super Learner		0.160	0.048	0.679	0.610, 0.747	0.613	0.675	0.691	0.898	0.818	0.964	0.701
Super Learner		0.156	0.073	0.701	0.632, 0.771	0.593	0.537	0.810	0.788	0.870	0.944	0.224
LASSO	0	0.159	0.051	0.681	0.611, 0.752	0.667	0.550	0.760	0.808	0.903	0.927	0.467
Ridge	0.29	0.157	0.067	0.699	0.630, 0.768	0.714	0.500	0.765	0.843	0.887	0.936	0.168
Elastic Net	0	0.159	0.055	0.684	0.613, 0.755	0.700	0.500	0.776	0.825	0.918	0.925	0.245
GBM	0.202	0.158	0.057	0.684	0.614, 0.753	0.579	0.657	0.716	0.833	0.904	0.900	0.515
Random Forest	0.126	0.161	0.039	0.677	0.605, 0.748	0.553	0.720	0.811	0.744	0.857	0.923	0.029
MARS (polymars)	0	0.170	-0.015	0.550	0.471, 0.629	0.579	0.783	0.781	0.875	0.889	0.853	<0.001
MARS (earth)	0.013	0.188	-0.121	0.646	0.570, 0.722	0.627	0.759	0.756	0.762	0.833	0.864	<0.001
Bayes GLM	0.368	0.158	0.060	0.690	0.619, 0.761	0.532	0.769	0.763	0.767	0.867	0.909	0.270
BAGGed CART	0	0.161	0.043	0.677	0.585, 0.730	0.605	0.649	0.735	0.829	0.884	0.894	0.312

Abbreviations: BAGGed CART, bootstrap aggregated classification and regression tree; CV, cross-validated; LASSO, least absolute shrinkage and selection operator; GBM, generalized boosted regression; GLM, generalized linear model (i.e., main effects logistic regression); MARS, multivariate adaptive regression splines

[a]Weights of the individual algorithms for the Super Learner estimate

[b]Cross-validated; i.e., computed in data omitted from algorithm training set.

[c]Means (proportions) with EED, in strata defined by cross-validated probabilities estimated from the algorithms. In well-calibrated models, the means should fall within the ranges of the strata.

FIGURE LEGENDS

Supplementary Figure 1. Heat map showing the relationship of serum amino acids, biogenic amines, amino acid metabolites, acylcarnitines, and sphingolipids by gut permeability (L:M ratio). L:M ratio is categorized in deciles. Abbreviations for lipid nomenclature and other metabolites are described in the methods section.

Supplementary Figure 2. Heat map showing the relationship of serum glycerophospholipids by gut permeability (L:M ratio). L:M ratio is categorized in deciles. Abbreviations for lipid nomenclature and other metabolites are described in the methods section.

Supplementary Figure 3. Cross-validated receiver operating curves of the relationship of the 14 significant serum metabolites with EED using Super Learner, cross-validated (CV) Super Learner, and nine other algorithms. Abbreviations: BAGGed CART, bootstrap aggregated classification and regression tree; CV, cross-validated; LASSO, least absolute shrinkage and selection operator; GBM, generalized boosted regression; GLM, generalized linear model (i.e., main effects logistic regression); MARS, multivariate adaptive regression splines

Supplementary Figure 1

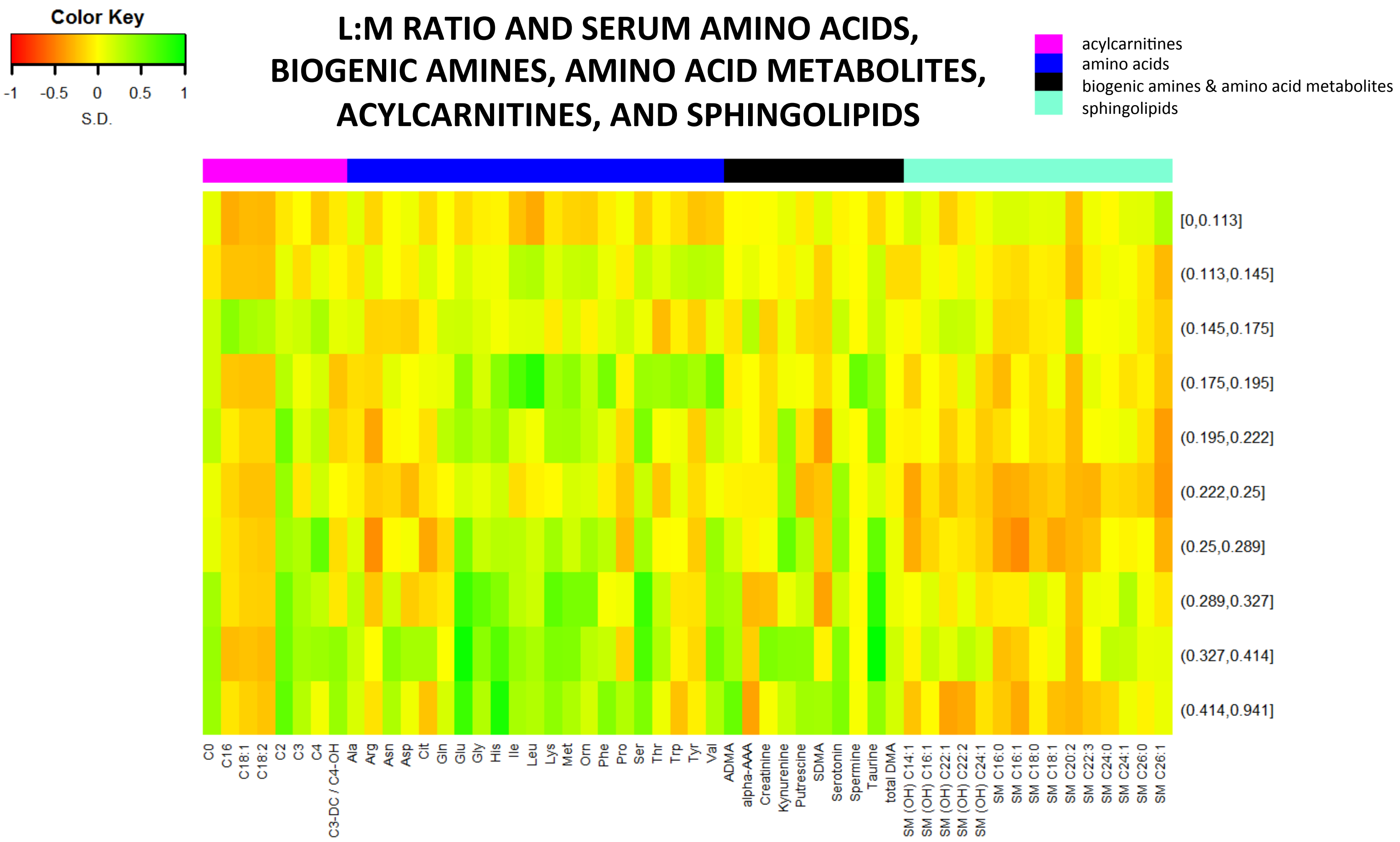

Supplementary Figure 2

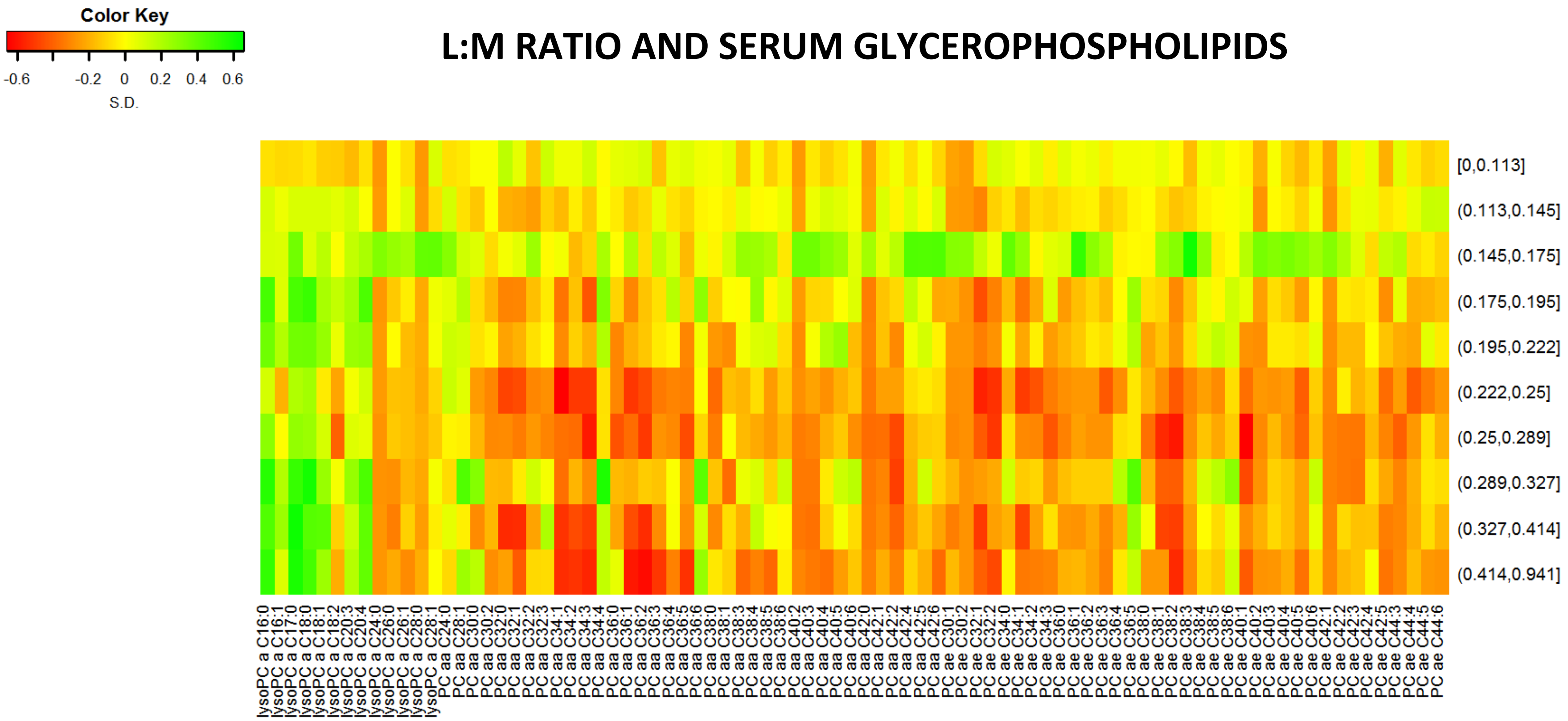

Supplementary Figure 3

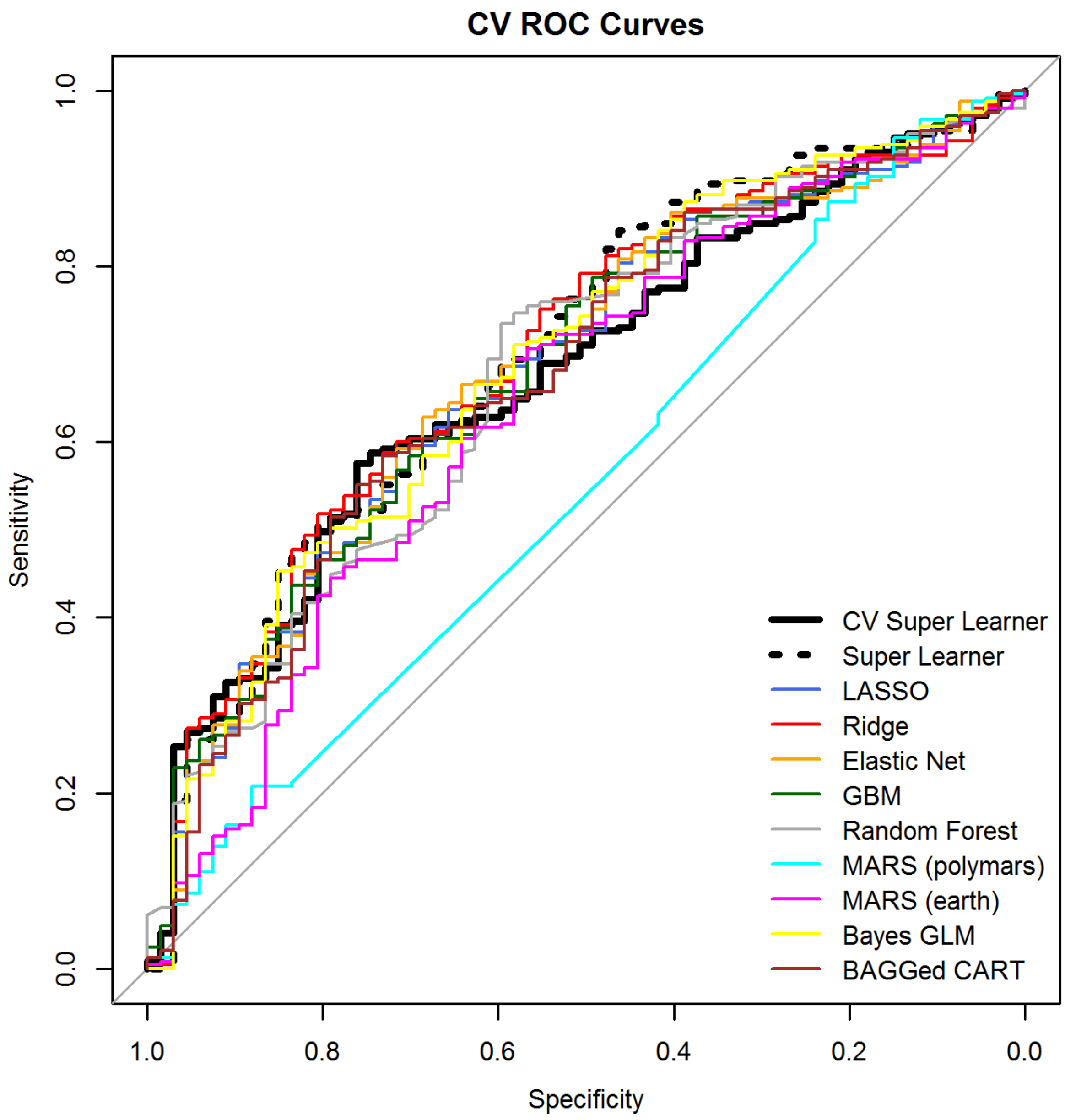

Supplement: Supplementary Information [file srep28009-s1.pdf]
